# P-1898. Evaluation of a Formal Curriculum for Infectious Disease Elective Students

**DOI:** 10.1093/ofid/ofaf695.2067

**Published:** 2026-01-11

**Authors:** Jessica R Newman, Charles Walde, Victoria Poplin

**Affiliations:** University of Kansas Medical Center, Fairway, Kansas; University of Kansas Medical Center, Fairway, Kansas; University of Kansas Medical Center, Fairway, Kansas

## Abstract

**Background:**

The University of Kansas School of Medicine adult Infectious Diseases (ID) 4^th^ year elective focused education on cases encountered on the wards, which can lead to gaps in content^2^. A comprehensive ID elective is important to medical student knowledge and can be a tool for recruitment into the field. The aim of this quality improvement project was to assess the impact of a structured curriculum on student’s ID knowledge and overall satisfaction with the rotation.

**Figure:**
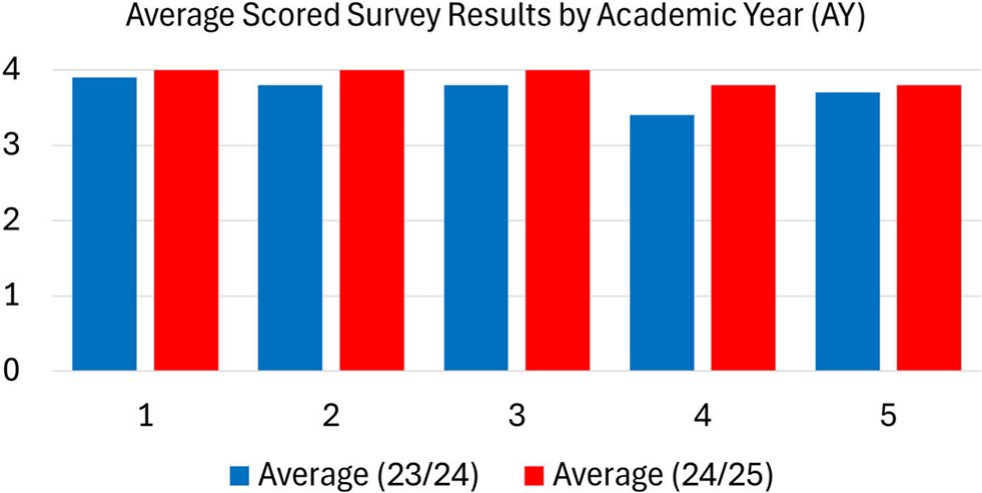
Average Scored Survey Results by Academic Year (AY) Student rotational survey scores for questions 1-5 for pre (23/24) versus post (24/25) intervention

**Figure:**
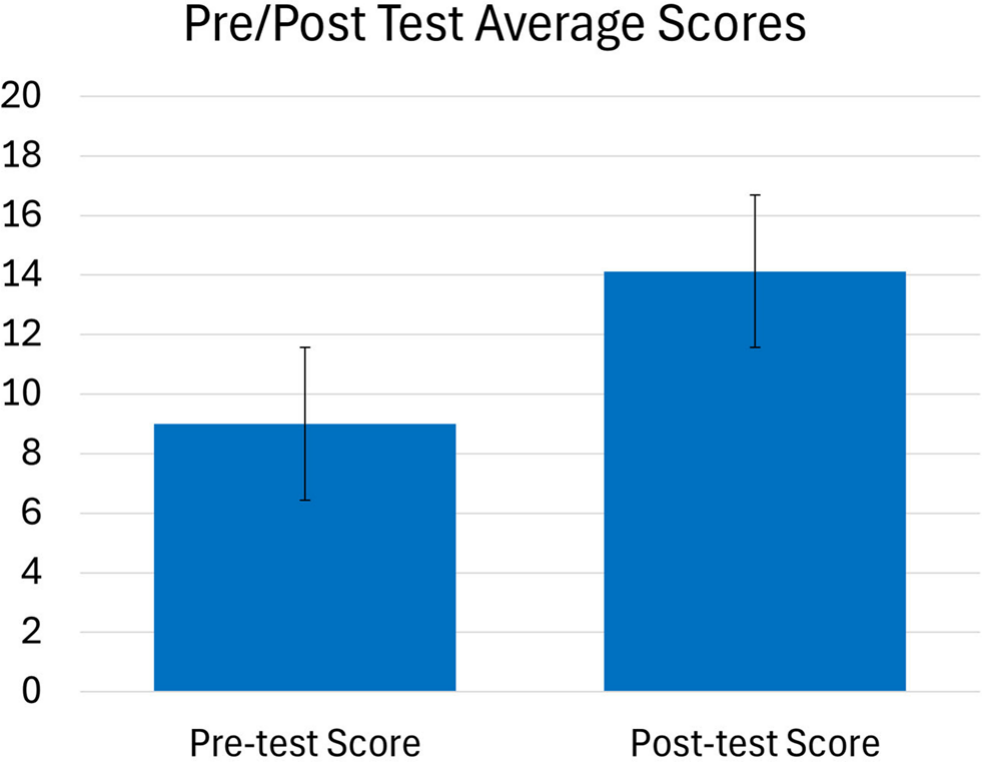
Pre/Post Test Average Scores Student pre and post-test scores on ID-related knowledge multiple-choice questions for 24/25 AY

**Methods:**

This single-center quasi-experimental study used pre/post surveys and tests to compare the impact of a revised curriculum. During the 2024-2025 academic year (AY), curriculum changes included an orientation, dedicated time to complete online modules, and coaching sessions. Satisfaction surveys and pre/post tests were administered to assess the curriculum changes on student perception of experience and ID knowledge. Faculty and fellows were evaluated for satisfaction & student preparedness.

**Figure:**
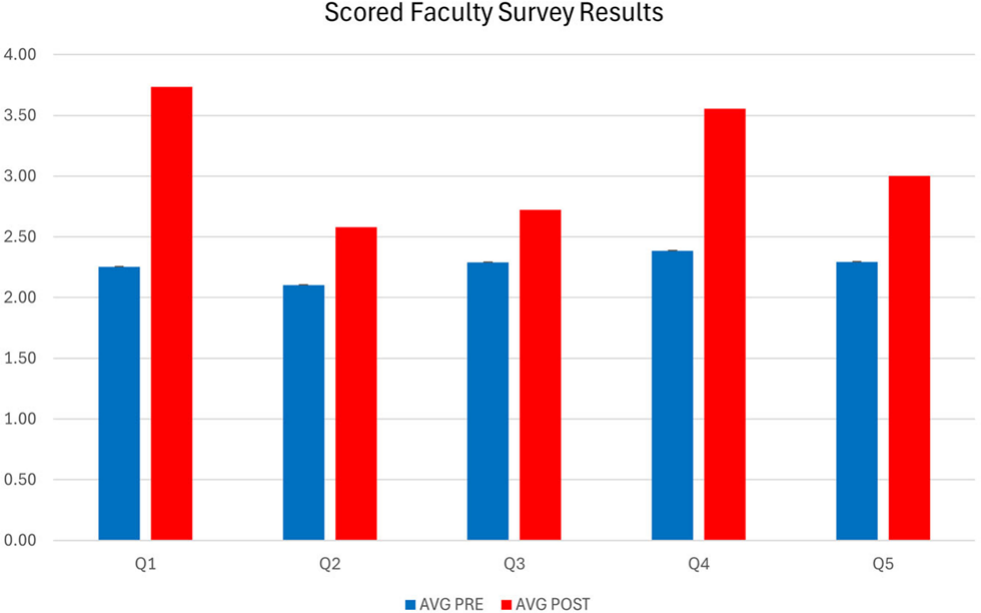
Scored Faculty Survey Results Pre (23-24) and Post (24-25) Faculty/Fellow Satisfaction Survey Results

**Results:**

All student survey questions 23-24 and 24-25 AY, responses were favorable, and there was a non-significant trend towards higher scores in the intervention group (Figure 1). On faculty/fellow surveys, there were statistically significantly higher scores on post intervention survey for student preparedness, optimal elective structure and adequate time to complete online modules (Figure 2).

Pre/Post Test Results: In AY 24-25 the average pretest score was 9.0 (+/- 2.0) and average post-test score was 14.4 (+/- 2.3). Using a student t-test there was a statistically significant difference in the pre versus post-test total scores (P value < 0.001, Figure 3). There was no available comparison data from 23-24 AY to compare.

**Conclusion:**

Pre-post test data did show a significant increase in average student ID knowledge, but it is unclear if this is a result of the curriculum changes. However, students, faculty, and fellows did report high satisfaction with the rotation and the. intervention was easily implemented with minimal cost. We are hopeful that the curriculum changes will interest more students in the elective, increase faculty excitement in teaching, and promote future recruitment.

**Disclosures:**

All Authors: No reported disclosures

